# Is biomedical research demand driving a monkey business?

**DOI:** 10.1016/j.onehlt.2023.100520

**Published:** 2023-03-03

**Authors:** Regina Kate Warne, Georgia Kate Moloney, Anne-Lise Chaber

**Affiliations:** School of Animal and Veterinary Sciences, The University of Adelaide, Adelaide, South Australia, Australia

**Keywords:** CITES, Conservation, Macaque, Medical/scientific research, Public health, Wildlife trade

## Abstract

For decades, animal models such as the macaque have been used in the advancement of human medicine and therefore have been subject to extensive trade globally. The sustained need of macaques for research necessitates assessment of the international trade and whether appropriate regulations are in place to safeguard animal welfare, public health and scientific integrity. In this study, we investigated the trade in live macaques (*Macaca fascicularis)* for commercial, scientific and medical purposes reported through the CITES Trade Database between 2000 and 2020 from selected countries. Discrepancies were evident in the data collected, particularly associated with the quantities of live animals reported by the exporting and importing countries. Of particular interest were the trade discrepancies reported between 2019 and 2020, wherein Cambodia significantly increased their exports of macaques whilst China, traditionally one of the largest suppliers of macaques, ceased all exports. Concurrently there were notable inconsistencies between the macaque trade permitted for export to the United States and the import quantity reported. Such findings suggest that the macaque trade requires more stringent monitoring in order to minimise potential illegal wildlife trade activity and reduce the risk of zoonoses or pathogen spill-over events. Therefore, increased regulation on a global scale is required to ensure that the supply of macaques is legitimate, supports quality research and does not provide an opportunity for future disease outbreaks to occur.

## Graphical abstract

**Figure:**
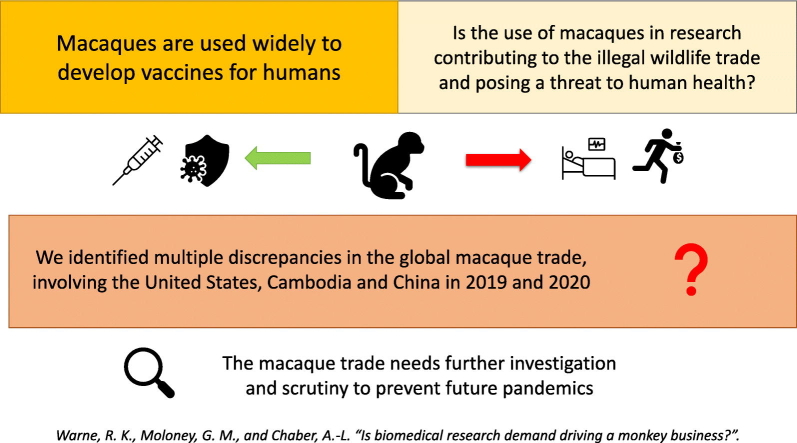

## 1 Introduction

Irrespective of increased public scrutiny, the global wildlife trade has continued to flourish to provide meat, traditional medicine, pets and products of cultural significance and status [1]. The legal wildlife trade must be conducted in accordance with relevant domestic laws, the Convention on International Trade in Endangered Species of Wild Fauna and Flora (CITES) and other international agreements and governing bodies [2]. Such regulations are in place to avoid the unsustainable trade of wildlife which can threaten public health [3] and ecosystem biodiversity [1]. As such, it has become increasingly important to regulate and monitor the trade of wildlife while discerning legal from illegal trade activity. Unfortunately, the distinction between illegal and legal trade has not only become blurred [4], but there is also increasing evidence of the legal trade being used as a loophole for the illegal trade [5,6].

An important aspect of the wildlife trade is the supply of animals and their products for scientific and medical research. Non-human primates (NHP) have long been the preferred animal model for biomedical research due to their phylogenetic relatedness and therefore anatomic and physiologic similarities with humans [7]. A popular taxon used in such studies is the macaque [5] for which there are 24 recognised species across the globe with varying conservation statuses [5,8]. The most popular species used for scientific research is the long-tailed macaque (*Macaca fascicularis*) which accounts for 10% of the annual revenue for all animals exported globally [9]. *Macaca* spp. have an important role in combating human infectious disease outbreaks as they have been widely utilised in the development of human vaccines [10] including AIDS/HIV [11], periodontitis [12] and most recently SARS-CoV-2 (COVID-19) [13].

However, the contribution of *M. fascicularis* to scientific research can be detrimental to the conservation of wild populations. The breeding and trade of macaques historically has been poorly regulated in South-East Asia, a large international supplier of macaques [14]. This can give rise to more wild macaques being captured than is sustainable as well as cause death or injury of the primates in the pursuit of capture [14]. To prevent overexploitation, permits are required for the trade of *M. fascicularis* due to their listing in CITES Appendix II [15,16]. In spite of this, the long-tailed macaque was reclassified as ‘endangered' in 2022 on the International Union for Conservation of Nature (IUCN) Red List. Major exporters of macaques between 2000 and 2020 included China, Laos, Mauritius, Cambodia, Thailand, Indonesia and Viet Nam, with their capacity to supply macaques fluctuating over time to complement global demand [14,17]. Traded macaques from these regions can be sourced from wild populations or produced in breeding facilities, either located within their habitat native habitat range (e.g. China, Mauritius, Cambodia, Viet Nam, Laos [8,18]) or outside their native range (e.g. US, Europe [17]). Macaques obtained from captive breeding facilities have been favoured since the early 1980s [19]. Motivations driving this change in preference differs among trading countries, but can include the increased demand for macaques for use in research, the heightened concern for conservation of wild populations, greater scrutinization of the health of traded animals and the increased demand for specific-pathogen free (SPF) animals for research [14,20]. SPF animals are of particular interest given the public health risk associated with the handling of macaques by human personnel. Primates can harbour zoonotic pathogens like B virus, *Mycobacterium* spp., simian foamy virus, hepatitis B virus and *Plasmodium* spp. Spill-over of zoonotic agents is enhanced given the macaque is closely related to humans [3,21] and these particular pathogens can lead to serious and potentially fatal infections in humans [22]. Therefore, legitimate, legal captive breeding of macaques not only helps to alleviate depletion of wild populations and promote their conservation, but also safeguards public health if done in accordance with current recommendations and legislative requirements.

The sustained use of animals for research calls for an assessment of both the legal and illegal global wildlife trade. In this study, we investigated the global trade of macaques for scientific, commercial, or medical purposes reported by CITES between 2000 and 2020. Based on our findings, we elected to examine the Cambodian macaque trade with greater granularity using data available from the CITES Trade Database. The aim of this study was therefore to critically assess the trade of macaques and comment on trade discrepancies reported and the potential public health implications.

## 2 Methods

Metadata was extracted from the open access CITES Trade Database [21] on 6 October 2022.

We queried for records of live macaques of all species, of any source, reportedly traded for scientific, commercial, or medical purposes between 2000 and 2020 inclusive. Only record of live macaques were assessed; the trade of other macaque specimens were not included. Each record is equivalent to one trade permit and includes the year the trade occurred, the number of animals traded by the exporter and by the importer, the source (wild-caught, captive born, captive bred), the exporting and importing country and whether the trade occurred directly or indirectly (i.e. if a transit country was involved). For definitions of trading terms, refer to Appendix A.

For an exporting country to be included in this study, the country was required to be either (1) a known major global producer of macaques [23] or (2) a country of South-East Asia given such countries are a large component of the geographic distribution of most macaque species [[17], [18], [20]].Therefore, exporting countries included Cambodia, Singapore, Thailand, Indonesia, Viet Nam, Timor-Leste, Brunei Darussalam, Philippines, Myanmar, Malaysia, Lao People's Democratic Republic, Israel, China and Mauritius [8,18,20,23,24]. All other countries of export were excluded given they had either negligible trade or were not the country of origin for the majority of trade reported to CITES, as observed during the preliminary reports screening. All importing countries were included. The database was accessed again on 1 January 2023 to identify any changes in records, however all data presented will be based on the search conducted in October 2022 unless otherwise specified. To supplement the most recent data, the Centre for Disease Control and Prevention (CDC) 2022 trade report was also assessed [25].

Major disease outbreaks of public health concern were identified based on the declared Public Health Emergencies of International Concern (PHEIC) by the International Health Regulation [26]. Data visualisation was conducted in R Studio (version 3) [27] using the packages ggplot2 [28] and networkD3 [29].

## 3 Results

The CITES database search yielded 1474 records of trade shipments fulfilling the requirements outlined above [30] and were therefore included in this study. Only 463 of these records displayed both the number of macaques exported and imported.

By far, the most commonly exported taxon was *Macaca fascicularis* (94.7%) and therefore this species will be the focus of this paper. Other taxa exported included *Macaca mulatta*, *Macaca nemestrina*, *Macaca arctoides*, *Macaca leonine* and *Macaca pagensis*.

**Fig. 1:**
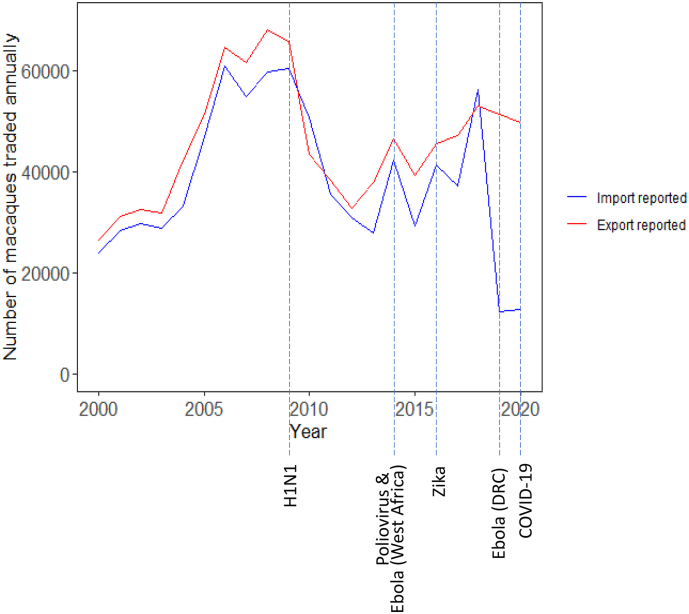
Global macaque trade relative to public health emergencies of international concern: The number of live macaques traded between 2000 and 2020 as reported by all importing countries (blue) and selected exporting countries (red; Cambodia, Singapore, Thailand, Indonesia, Viet Nam, Timor-Leste, Brunei Darussalam, Philippines, Myanmar, Malaysia, Lao People's Democratic Republic, China, Mauritius and Israel) relative to the declaration of public health emergencies of international concern (PHEIC) [26]. PHEICs declared between 2000 and 2020 include Influenza A (H1N1; declared April 2009), Poliovirus (declared May 2014), Ebola of West Africa (declared August 2014), Zika virus (declared February 2016), Ebola of the Democratic Republic of Congo (Ebola DRC; declared July 2019) and SARS-CoV-2 (COVID-19; declared January 2020) [26]. (For interpretation of the references to colour in this figure legend, the reader is referred to the web version of this article.)

The macaque trade does not appear to follow a particular trend but continues to change over time (Fig. 1). Peaks in macaque export appear to correspond with declarations of public health emergencies in 2014 (poliovirus and Ebola of West Africa) and 2016 (zika virus), but trade seems to decrease in the year following these declarations. Increases in export do not appear to occur for the remaining PHEICs declared since 2007. Additionally, the importing and exporting countries annual reported trade consistently do not match, with the difference between the quantities reported being greatest in 2019 and 2020 (Fig. 1 and Appendix D). Previously, the largest difference between import and export quantities occurred in 2017, however the difference reported in both 2019 and 2020 is approximately four times greater than this value.

### 3.1 Main importers and exporters

**Fig. B.1:**
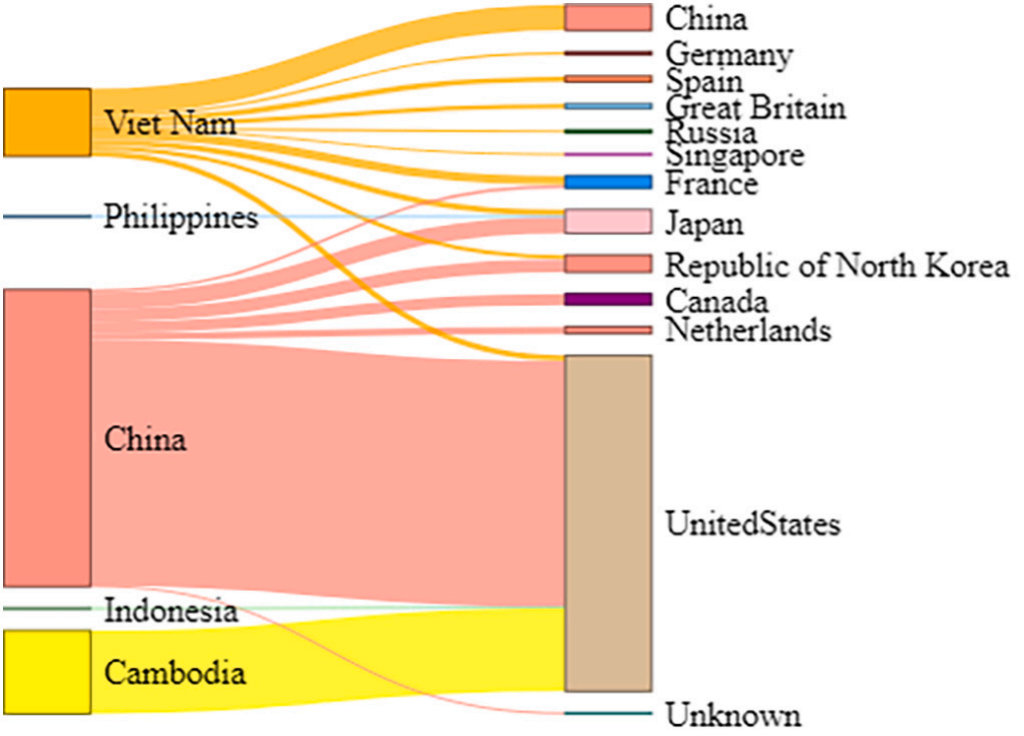
2018 Global Macaque Trade: The net export of live macaques traded in 2018 as reported by selected exporting countries (left; Cambodia, Singapore, Thailand, Indonesia, Viet Nam, Timor-Leste, Brunei Darussalam, Philippines, Myanmar, Malaysia, Lao People's Democratic Republic, China, Mauritius and Israel) to any importing country (right).

**Fig. B.2:**
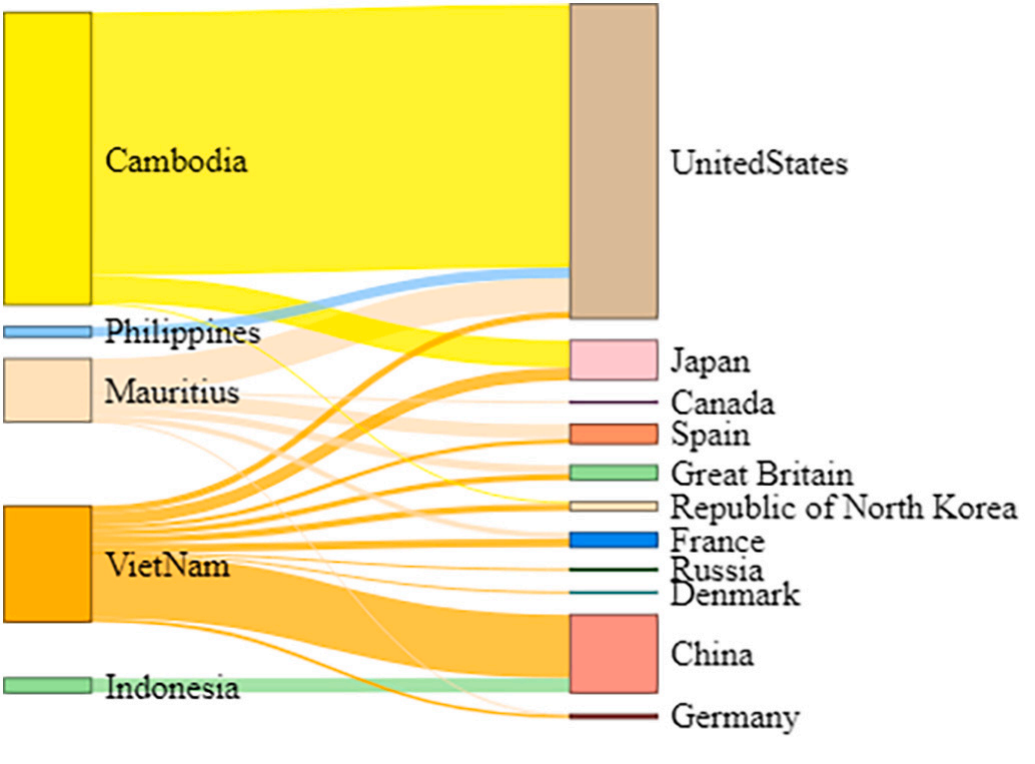
2019 Global Macaque Trade: The net export of live macaques traded in 2019 as reported by selected exporting countries (left; Cambodia, Singapore, Thailand, Indonesia, Viet Nam, Timor-Leste, Brunei Darussalam, Philippines, Myanmar, Malaysia, Lao People's Democratic Republic, China, Mauritius and Israel) to any importing country (right).

**Fig. B.3:**
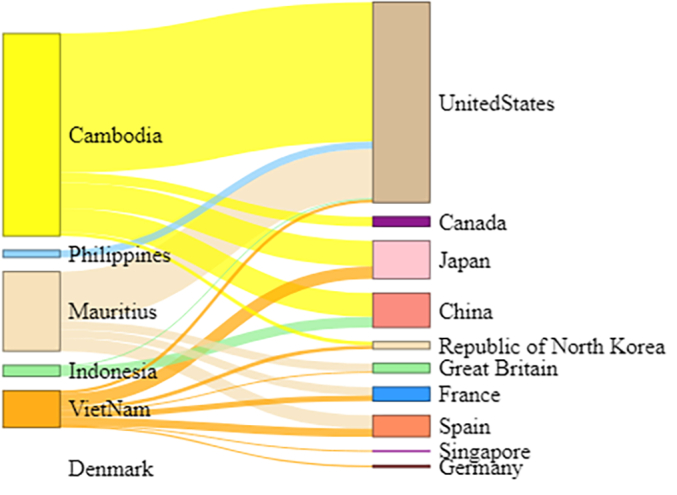
2020 Global Macaque Trade: The net export of live macaques traded in 2020 as reported by selected exporting countries (left; Cambodia, Singapore, Thailand, Indonesia, Viet Nam, Timor-Leste, Brunei Darussalam, Philippines, Myanmar, Malaysia, Lao People's Democratic Republic, China, Mauritius and Israel) to any importing country (right).

China was the largest exporter of macaques between 2000 and 2018 inclusive, accounting for between 32.5% to 66.0% of the total number of macaques directly traded, followed by no reported exports in 2019 and 2020 (Appendix B, Fig. B.1). Hereafter, Cambodia was the largest exporter of macaques, contributing to 59.0% of all macaques traded directly and indirectly in 2019 and 2020 (Appendix B, Fig. B.2, Fig. B.3). Prior to 2019, the greatest proportion of macaques Cambodia contributed to export was 24.7% in 2006.

The US was the largest importer of macaques, accounting for between 41.7% and 70.1% of the total annual trade between 2000 and 2018 inclusive. However, this was followed by no reported trade into the US in 2019 and 2020 on the CITES Trade Database (Appendix C, Figure C.1). When the database was re-accessed, reported import by the US had become available for 2019, but not for 2020 (Appendix C, Figure C.2) while the 2021 CDC reported import of macaques to have occurred in both 2019 and 2020 [25]. Other major importers for the 2000 to 2020 period included France (up to 17.1%), Great Britain (up to 15.9%), Japan (up to 37.9%) and China (up to 33.5%).

### 3.2 Cambodian macaque trade

**Fig. 2:**
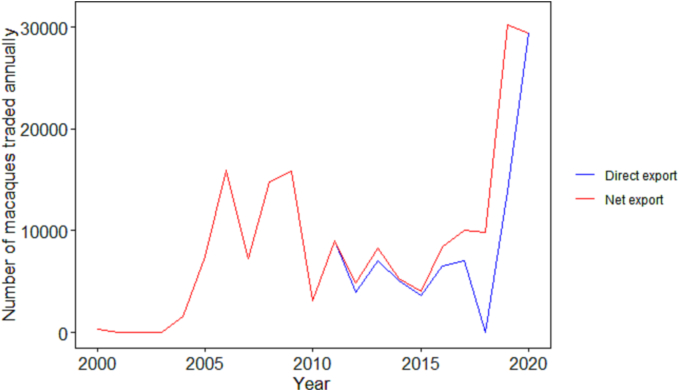
Direct and indirect Cambodian macaque export: Comparison of the number of live macaques annually exported directly from Cambodia to an importing country (blue) and the net export of live macaques from Cambodia including both direct and indirect export (red) from 2000 to 2020 for scientific, medical or commercial purposes. (For interpretation of the references to colour in this figure legend, the reader is referred to the web version of this article.)

For the majority of the 2000 to 2020 period, indirect export was either minimal or absent in Cambodia's annual macaque trade (Fig. 2). However, for 2017 to 2019, there was a marked increase in the indirect trade reported (Fig. 2) as the proportion of Cambodian macaque trade responsible for indirect trade increased from 30% in 2017 to 100% in 2018. Cambodia reported 1008 of their 185,875 total macaque export for the 2000 to 2020 period as wild-caught, accounting for 100% of the 2000 trade, 2.2% of 2020 and 0% of all remaining years which were instead either captive-bred or captive-born (Appendix D, Table D).

## 4 Discussion

### 4.1 The global macaque trade

The macaque trade for research purposes is becoming increasingly lucrative [5]. Reported prices for macaques fluctuate through time, ranging from $2800 [5,9] to $5000 USD [Chaber *pers. communication*], with prices rising as the supply reduces. With the low availability of macaques seen today [31], an individual macaque can be sold for between $20,000 and $24,000 USD [32]. These high prices may incentivise increased production of captive-bred macaques, with the COVID-19 era also sparking an increased need for both captive-bred [33] and SPF non-human primates [7] for research. Irrespective of whether they have such SPF status, all non-human primates used for biomedical research must have documentation supporting their health status and evidence of pathogen screening [33,34].

The macaque trade is dynamic with fluctuations in trends over time (Fig. 1), many of which warrant further investigation. While sharp peaks are observed, it is difficult to attribute all increases in the global macaque trade to a specific inciting cause. Macaques have been used for research in many scientific fields including neuroscience [35] and dentistry [12], but it seems less likely that these applications would be responsible for the large increases in the global trade observed. Vaccine development during disease outbreaks would seem the most reasonable explanation given the importance of macaques in medical research [10]. However, increases in the macaque trade do not appear to consistently coincide with PHEICs declared by the International Health Regulations (Fig. 1) [21]. Since 2000, up to 66.0% of the total export of macaques was from China, however in 2019 and 2020 the Chinese macaque trade decreased by 96% [5]. China officially banned wild animal trade on January 26, 2020 in light of the COVID-19 outbreak and concerns for public health and national security [31,36,37], following the earliest confirmed cases of COVID-19 in China in December 2019 [38]. As such, neither can account for the 96% drop in Chinese macaque exports which was seen in 2019. Therefore, it is unclear what the underlying driver of this shift was and, given it does not coincide with any pandemic declarations, the true reason requires further investigation.

### 4.2 Trade discrepancies

There are major discrepancies between the trade reported by exporting and importing countries (Fig. 1). The difference in reported trade averaged 7495 macaques throughout the 2000 to 2020 period and peaked at 39,162 macaques in 2019 (Appendix E). The CITES Trade Database Guide [39] lists multiple reasons for why this can occur, including: lower actual trade than what was initially permitted, mortality during transit and differing years of permitting and shipment [39,40]. It could also be a simple lack of reporting as not all CITES Parties report their import of Appendix II species, such as the macaque [40]. Further investigation revealed that the large discrepancy observed in 2019 and 2020 (Fig. 1) was likely attributed to this lack of reporting to CITES. From 2000 to 2018, US imports largely mirrored the exports reported by the exporting countries (Appendix C, Figure C.1). However, in 2019 and 2020 the US reported no macaque imports even though CITES permits were issued for over 25,000 macaques to be exported to the US (Appendix C, Figure C.1). When the database was re-accessed on 1 January 2023, the number of macaques imported by the US had become available for 2019 and corresponded closely to what was reportedly exported to the US by exporters. According to the CDC [25], the US imported 32,439 macaques in the 2019 financial year which, again, nearly reflects that reported by exporters to CITES. However, the same report stated that 24,879 macaques were imported by the US, nearly 4000 less than what was reportedly exported to the US by the CITES Trade Database, which is not unusual based on trade occurring in previous years. Regardless, it is critically important to determine whether this has occurred because the US delayed reporting to CITES, or due to a delay in CITES processing submitted reports. We therefore call for a response from both CITES and the US to answer why there was an almost three-year delay before CITES reports were published, while other importers had already submitted their 2019 reports. How promptly the US had previously submitted reports is not publicly available and it is important to know the usual expected delay in reporting. The scale of the US macaque trade and therefore the number of permits required to be processed should be considered a reason for the delay. Likewise, the impact of the COVID-19 pandemic upon staffing and routine procedures is another possibility. However, whether these could account for reports not being available until now is debatable, especially since the CDC reported the imports more than a year before they became available on the CITES Trade Database. It is unclear whether the US had always intended to submit their reports for 2019 or if this was only due to the recent spotlight on trade of macaques with the US. In November 2022, US authorities indicted eight individuals, including a Cambodian Government official, for suspected macaque trafficking to the US. If this incited the reports to be submitted, or whether it was truly a delay, should be investigated. Ultimately, it is crucial that the annual trade of macaques can be quickly assessed especially if it concerns the single greatest importer of macaques.

### 4.3 Breeding and export capacity of Cambodia

Despite losing China as a major exporter, global macaque exports persisted as other countries increased their trade capacity to supply global demand, even if they did not historically have the breeding infrastructure to match that of China [5]. One such country was Cambodia which increased their net export from 10,000 macaques in 2018 to 30,000 macaques in 2019 and 2020 (Fig. 2). Due to this considerable increase in trade, we selectively investigated Cambodia's trade data and breeding capacity with greater scrutiny.

To supply the 30,000 macaques reportedly exported in 2019 and 2020, we calculated that Cambodia would require at least 98,000 macaques (Appendix F.1 and Fig. F) to be housed across the 6 breeding facilities presumed to be operating at that time [41]. It is unlikely, even in well-run enterprises, that each mating yields a successful pregnancy carried to term with no juvenile mortality [42,43]. Based on previously reported stillborn and neonatal fatality rates in captive-bred *M. fascicularis* [44], the required capacity across these Cambodian breeding facilities would need to have increased to at least 102,948 macaques to produce 30,000 macaques for export in 2019 (Appendix F.2 and F.3). However, the rates used in our calculations are only the minimum expected and are likely to vary depending on parity and management. Therefore, in accounting for replacement stock production, poor management, greater parity and losses from infertility, this number increases further. Animals also need to be housed until they can be traded, usually at 2–5 years old [19], and consequently production and capacity would have needed to increase in 2017 to accommodate for the 2019 surge. With these additional considerations, the theoretical size of the farms far exceeds the conservative 98,000 calculated, as well as the less conservative estimate of 102,948. From 2010 to 2014, the six Cambodian breeding facilities collectively housed 81,926 macaques (breeding stock and offspring) [41] and the total number of breeding females per year did not exceed 26,306 individuals. These 81,926 individual macaques enabled 26,187 macaques to be permitted for export from Cambodia across the 2010 to 2014 period [Warne *pers. communication*]. Cambodia has therefore somehow managed to increase their macaque production rates from 81,926 over a four-year period to at least 98,000 in a single year. Concurrently, the number of breeding females would have needed to increase from 26,187 to more than 60,000 by conservative estimates.

In order to achieve the increase in trade, macaques could have been supplied through four possible sources: increasing legitimate production throughout breeding farms, wild-capture, non-accredited breeding farms or sourcing through other countries (imports or smuggling) [45,46]. Cambodia has never reported any live macaque imports and recent interviews with a Ministry of Agriculture, Fisheries and Forestry (MAFF) representative claim, “No captures [of macaques] from the wild has been allowed” since 2014 [41]. This is mostly mirrored in CITES reports which suggest that between 2014 and 2019 all Cambodian macaques traded were ‘captive-bred' or ‘captive-born’, however 2.2% of macaques exported in 2020 were ‘wild-caught’. Nonetheless, in the absence of external sources of macaques and to maintain legitimate production, facilities are dependent on their own breeding stock. Cambodia has historically been incapable of producing second generation offspring macaques [19,47], therefore increasing their production capacity legally seems unlikely. Therefore, the current production capacity of macaque breeding facilities needs to be fully investigated to determine the role, if any, of satellite farms, wild-capture and smuggling.

### 4.4 Illegal trade supplementing the legal macaque trade

Previous work has identified a positive correlation between legal imports and illegal seizures of wildlife, to the extent that the legal trade must act as a means for the illegal trade to continue [5,6,14,48]. Cambodia has a known history of unlawfully breeding and trading macaques [46,47,49]. They have previously failed to adhere to national regulations regarding wild macaque capture [1,47], misclassed traded animals as ‘captive-bred' [47] and have been involved in macaque-laundering schemes with Laos and Viet Nam [46,49]. The Cambodian macaque trade is also the subject of current investigations on an international scale. In 2022, the MAFF Director of Wildlife and Biodiversity and seven other individuals were indicted by US authorities under suspicion of trafficking wild-caught macaques as ‘captive-bred' [50]. The case outcome is yet to be determined, but if the single greatest global importer of macaques risks their continued supply of animals to flag potential illegitimacies of trade, we must ask whether there is reason for concern.

Cambodia is also the main source of macaques for indirect trade, which means that Cambodian macaques are exported to a transit country (e.g. Thailand) before being re-exported to another country. In 2018, Cambodia reported no direct trade of macaques but was responsible for supplying nearly 10,000 live macaques through the indirect trade (Fig. 2). Similarly, more than 15,000 of the 30,000 macaques exported by Cambodia in 2019 were as a result of indirect trade (Fig. 2) Since an importing country could directly source their macaques from Cambodia, one may suspect indirect trade activity serves as a means for macaque laundering. Given the scale of this potential laundering, complacency and/or corruption at all levels of trade may be required and must be further investigated.

### 4.5 Public health concern

Both the legal and illegal macaque trade can increase the risk of zoonoses or novel emerging infectious diseases, many of which could result in disease outbreaks. It is therefore important to consider both its scale and whether the practices align with current recommendations. The wildlife trade increases opportunities for pathogen spill-over events due to increased contact at the human-wildlife interface, where direct contact can occur at any point in the macaque supply chain including capture, rearing, transport and in research [[51], [52], [53], [54]]. Transmission risk is further increased as animals traded are often stressed, malnourished and maintained in unhygienic conditions with high stocking densities, such as that suspected in the Cambodian macaque trade [[51], [52], [53], [54]]. Meanwhile, despite the provision of accompanying documentation, the legitimacy of the health and disease screening certificates should be scrutinised to further safeguard public health and suitability for medical research. Unfortunately, the reports from MAFF's inspections of Cambodian breeding facilities are not publicly available and independent organisations have been denied access to the sites since before the COVID-19 pandemic [41]. Therefore, international authorities are unable to determine the legitimacy of macaque breeding, the threat these activities pose to society, nor the appropriate mitigation procedures which need to be implemented to prevent both sporadic infections, epidemics and pandemics.

### 4.6 Recommendations

Any disparity in the wildlife trade will be filled by other suppliers where a product is in high demand. Therefore, halting the Cambodian trade would only provide an opportunity for other countries to take its place and potentially continue a cycle of illegitimate trade. Therefore, we recommend stricter law enforcement strategies locally and globally with auditing of breeding facilities by national and international teams and regular monitoring of the trade reported to CITES, such that any suspicious activity can be promptly investigated. Traded animals should be randomly screened to ascertain provenance (e.g. via forensic technology) and SPF status at international borders or quarantine facilities upon entry. Ethics committees of research institutions should require proof of the breeding facility audit before permitting the import of animals for research. Lastly, it is the responsibility of the entire research team to not participate in suspicious trade, including refraining from the purchase of cheaper animals which are more likely to be illegitimate.

## 5 Conclusion

This study has highlighted various aspects of the global and Cambodian macaque trade that warrant further investigation. It should be determined why there were significant changes in the global macaque trade evident from 2019 including the delayed reporting in the US, China discontinuing their macaque exports and Cambodia increasing their macaque exports and indirect trade. While it would be easy to attribute this to COVID-19, WHO did not declare an ‘outbreak’ until January 2020 [38]. Therefore, understanding whether these changes share a stimulus, and how many are a direct consequence of another impetus, is vital in furthering our understanding of the macaque trade.

Additionally, the CITES Trade Database is freely accessible to the public, meaning anyone could ascertain these trends in the macaque trade. However, it has taken three years for anyone to flag any issues or discrepancies and therefore three years of missed opportunity to investigate and potentially correct any wrongdoing. This calls to question just how sustainable the CITES system is in regulating wildlife trade activity if the numbers reported are incomplete and not sufficiently analysed.

The global trade of macaques does not seem to be diminishing given the sustained demand by researchers, therefore there is a greater need now than ever to ensure that any breeding or trade in macaques is sustainable and legitimate. While the risks can be mitigated by improving regulation of breeding facilities, imposing veterinary checks and instigating biosecurity [52], the preservation of public health demands that any protocols implemented be prioritised, thorough and allow for timely responses to threats. It is crucially important the scientific communities enter this discussion and determine if, by racing to provide models for human medicine and vaccine development, the wildlife trade is instead paving the way for the next pandemic to occur.

## CRediT authorship contribution statement

**Regina Kate Warne:** Methodology, Investigation, Writing – original draft, Writing – review & editing, Visualization. **Georgia Kate Moloney:** Conceptualization, Methodology, Writing – review & editing, Visualization. **Anne-Lise Chaber:** Conceptualization, Methodology, Writing – review & editing, Supervision.

## Declaration of Competing Interest

All authors declare that we have no conflicts of interest.

## Figures and Tables

**Fig. C:**
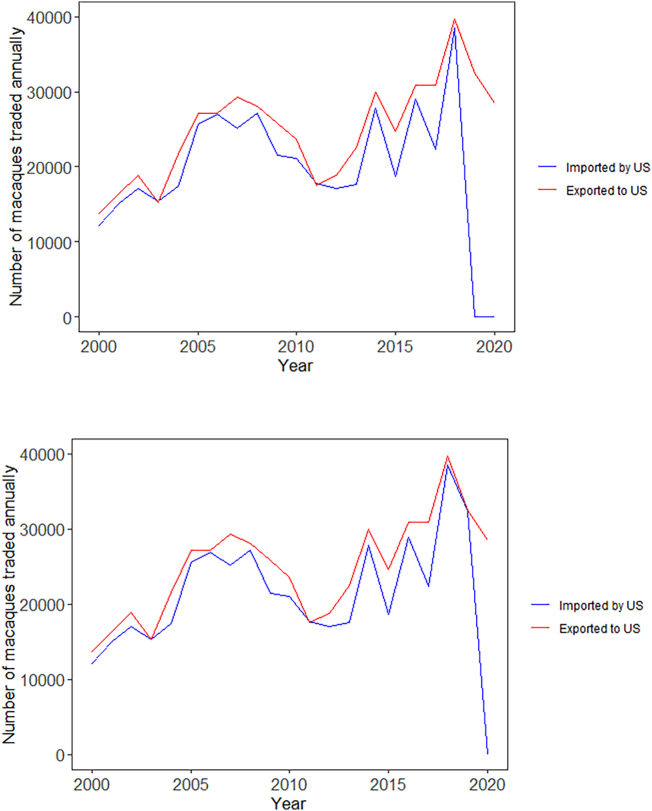
Trade to the US as per exporter and importer: Comparison of the gross number of live macaques traded to the US annually as per reports to CITES from 2000 to 2020 between the selected by the exporting countries (blue; Cambodia, Singapore, Thailand, Indonesia, Viet Nam, Timor-Leste, Brunei Darussalam, Philippines, Myanmar, Malaysia, Lao People's Democratic Republic, China, Mauritius and Israel) and the US, as reported on 6 October 2022 (1) and on 1 January 2023 (2) (For interpretation of the references to colour in this figure legend, the reader is referred to the web version of this article.)

**Table D:** The collated export of live macaques from Cambodia from the years 2000–2020 inclusive, from any source and for commercial, medicine, or scientific purposes. 2020 also had 360 macaques traded as ‘D' (bred in captivity for commercial purposes) not included in this table.

Year	Export reported (number of specimens)	Net Export (number of specimens)	Wild-caught (number of specimens)	Captive-bred (Number of specimens)	Captive-born (number of specimens)
2000	360	360	360	0	0
2001	0	0	0	0	0
2002	0	0	0	0	0
2003	0	0	0	0	0
2004	1590	1590	0	1590	0
2005	7430	7430	0	7430	0
2006	15,990	15,990	0	15,990	0
2007	7280	7280	0	5480	1800
2008	14,820	14,820	0	0	14,820
2009	15,860	15,860	0	0	15,860
2010	3095	3095	0	0	3095
2011	9050	9050	0	6000	3050
2012	3930	4822	0	220	4602
2013	7039	8259	0	0	8259
2014	5081	5241	0	0	5241
2015	3661	4084	0	0	4084
2016	6567	8349	0	165	8184
2017	7025	10,041	0	2264	7777
2018	0	9854	0	9854	0
2019	13,922	30,284	0	30,284	0
2020	29,466	29,466	648	19,269	2160

**Table E:** The number of live macaques traded between 2000 and 2020 for scientific, medical, or commercial purposes, from any source, as reported by all importing countries and selected exporting countries (Cambodia, Singapore, Thailand, Indonesia, Viet Nam, Timor-Leste, Brunei Darussalam, Philippines, Myanmar, Malaysia, Lao People's Democratic Republic, China, Mauritius, or Israel), and the difference between reported quantities. The year in which the minimum, maximum or median was reported has also been included.

	Minimum macaques traded (year)	Maximum macaques traded (year)	Mean macaques traded	Median macaques traded (year)
Import (all countries)	12,205 (2019)	60,939 (2006)	38,269	35,619 (2011)
Export (all countries)	26,583 (2000)	68,122 (2008)	45,764	45,584 (2016)
Difference in number of macaques traded (year)	−7230 (2010)	39,162 (2019)	7495	4256 (2016)

**Fig. F:**
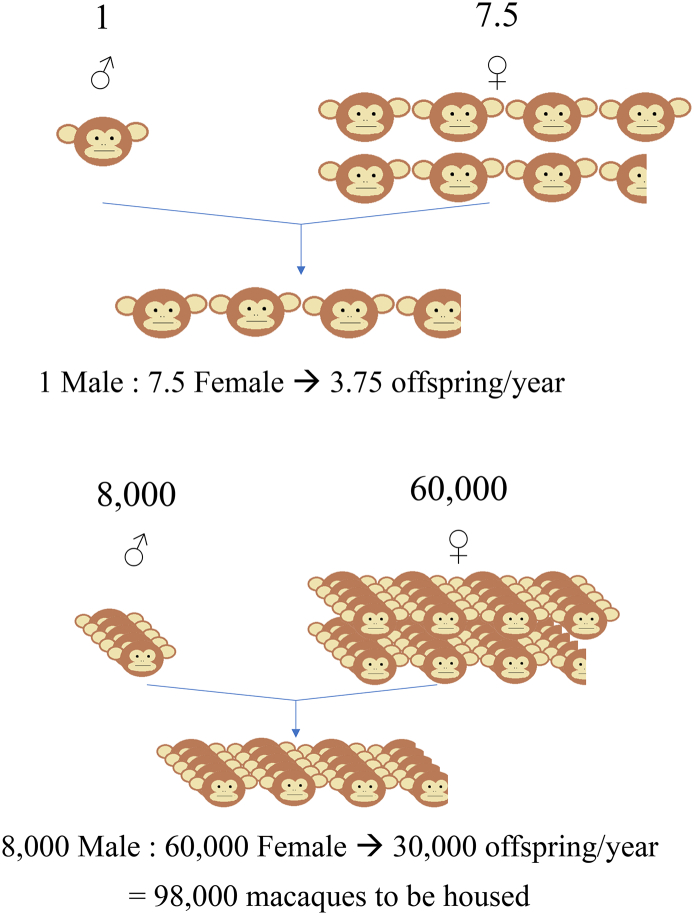
Estimated macaque breeding production: The theoretical yield and capacity for a macaque-breeding facility. A female macaque produces a single offspring approximately every two years (165 days gestation and 18 months between births) [19] and the joining ratio of macaques is approximately 1 male for every 7.5 females [55]. Therefore, to attain 30,000 macaques required for export in a single year, at least 98,000 macaques would need to be housed in breeding facilities across Cambodia.

## Data Availability

There is a datalink provided in manuscript references

## Appendix A Definitions

Source: as reported by the exporting country, refers to the original source of the species traded. In this study, relevant sources include captive bred (C), captive born (F; F1 or subsequent generations), and wild-caught (W).

Purpose: the intended use for the species traded. In this study, relevant sources include medical (M), scientific (S), and commercial (T).

Countries/parties: CITES parties involved in the trade of the species. Referred to by a two-letter ISO code, as per the CITES Trade Database Guide Version 9 [39]. Important codes include KH (Cambodia), US (United States of America), CN (China), JP (Japan), GB (Great Britain), and FR (France), among others.

Direct trade: the species are traded from the country of origin to the importer.

Indirect trade: the species are traded from one country to another and then re-exported to a third party [39].

## Appendix F Cambodian breeding facility capacity calculations

### F.1 Conservative estimate

Joining ratio = 1 male: 7.5 females [55].

Approximate production rate per female = 1 offspring every 2 years (165 days gestation and 18 months between births) [19].

∴ 1 male: 7.5 females ➔ 3.75 macaques per year.

In order to attain 30,000 offspring for export:

$\frac{30000}{3.75}=8000\;$breeding males required.

8000 × 7.5 = 60,000 breeding females required.

8000 + 60000 + 30000 = 980000 individuals to be housed.

### F.2 The number of births required to compensate for stillborn and juvenile mortality

Minimum neonatal fatality rate = 4.0% [44].

Average stillborn rate = 2.9% [44].

0.94 × 0.95 × *a* = 30000 macaques available for export.

where a = the total number of births

$$a=\frac{30000}{0.95*0.94}$$

*a* = 33595 births required to attain 30,000 macaques for export.

### F.3 Less conservative estimate (accounting for stillborn and juvenile mortality)

33,595 births required.

$\frac{33595}{3.75}=8959$breeding males required.

8959 × 7.5 = 67190 breeding females required.

67190 + 8959 + 30000 = 106149 individuals to be housed.
